# Does Spore Ultrastructure Mirror Different Dispersal Strategies in Mosses? A Study of Seven Iberian *Orthotrichum* Species

**DOI:** 10.1371/journal.pone.0112867

**Published:** 2014-11-20

**Authors:** Nagore G. Medina, Belén Estébanez

**Affiliations:** Departamento de Biología (Botánica), Facultad de Ciencias, Universidad Autónoma de Madrid, Campus de Cantoblanco, C/Darwin 2, Madrid, Spain; University of Vigo, Spain

## Abstract

Most mosses have xerochastic dispersal (i.e., they open their capsules when conditions are dry), which is thought to favor long-distance dispersal. However, there are several species that use a hygrochastic strategy: spores are dispersed when conditions are wet. The significance of this strategy in the Mediterranean region is unknown. In this study, we explored whether ultrastructural features related to differences in spore resistance may explain these different strategies of spore dispersal. To this end, we examined the ultrastructural features of the spores of seven closely related species in the moss genus *Orthotrichum*. These species all grow as epiphytes in sub-Mediterranean forests, and the group includes both xerochastic and hygrochastic members. First, we found that the spore wall layers exhibit several features previously undescribed in mosses. Second, we discovered that there are only subtle differences in spore ultrastructure with regards to spore wall thickness, the degree of plastid development, or the storage substances used. We suggest that the hygrochastic dispersal in mosses from Mediterranean environments might be related to a safe-site strategy, rather than to drought avoidance, and we underscore the necessity of conducting spore ultrastructural studies on a greater number of bryophyte species.

## Introduction

In bryophytes, spores play a crucial role in dispersal and in the establishment of new populations. During dispersal, spores may have to endure harsh conditions (e.g., drought, ultraviolet light, extreme temperatures), and thus spore resistance appears to be a major factor affecting the dispersal range of many bryophyte species. This idea has been experimentally tested in the Southern Hemisphere, where widely distributed species of both liverworts [1] and mosses [2] produce spores that are more resistant than those of species with constrained distributions. However, it remains unclear if different spore features, namely ornamentation, wall stratification, and cytological characters, reflect different dispersal strategies.

Since bryophyte spore ornamentation is much simpler than spermatophyte pollen ornamentation, transmission electron microscope (TEM) studies of spores have attracted little attention. Nevertheless, there are several studies showing the paramount importance of spore ultrastructure. In systematics, for instance, examinations of sporoderm stratification have allowed the main bryophyte lineages to be successfully resolved [3]. Mosses (Div. Bryophyta) have been shown to be the only division where a true perine is developed. In the quest to characterise the earliest land plants, the bryophytic nature of ancient palynomorphs is considered because of their multilamellar exine, which, in extant plants, have exclusively been observed in liverworts (Div. Marchantiophyta) [4].

Spore longevity and resistance to the stressful conditions they experience during dispersal and establishment vary substantially in non-vascular land plants [5]–[7]. This variability is often related to cytological characteristics [8], [9]. In pteridophytes, species are frequently grouped as presenting “green” or “non-green spores”, which differ in plastid development and sporoderm thickness. Non-green spores, with thicker spore walls and reduced plastids, are known to show delayed germination and longer viability [8].

Also in bryophytes, “green spores” have been found to be more sensitive and able to germinate immediately [10]. Even within the same genus, spores vary greatly in sporoderm thickness and plastid development, as in *Grimmia* [11]. Nevertheless, as only a few species have been examined (see e.g. [11]–[15]), the importance of variation in spore ultrastructure across the bryophytes is unknown, and it is therefore difficult to assess how systematic affinities or ecological constraints contribute to morphological diversity.

Moss spore dispersal usually occurs under dry conditions (xerochastic dispersal), thus maximizing dispersal distance. However, the reverse phenomenon (hygrochastic dispersal) has also been observed. The mode of capsule opening is a constant feature for any given species. It is a purely mechanical process that depends on the hygroscopic responses of the capsule mouth. Thus, its opening strategy type can easily be determined by observing a capsule's behavior when water is added or during drying.

Not surprisingly, the hygrochastic strategy is common in species from humid, tropical environments [16], where the efficiency of xerochastic dispersal would be reduced [17]. Interestingly, some Mediterranean species also resort to hygrochastic dispersal, a strategy whose significance in dry climates remains unknown and merits further research. A plausible explanation involves spore tolerance of harsh environmental conditions during long-distance dispersal. If spores dispersing under wet conditions are less tolerant, a hygrochastic strategy could be advantageous. Even if their ability to disperse far from their parental sporophyte is limited, these spores would benefit by avoiding the severe drought and intense irradiation that are typical of the Mediterranean climate. Van Zanten's experiments in the Southern Hemisphere [1], [2] partially support this hypothesis, as they provide evidence for a close relationship between the dispersal distance and the ability to resist stress during transportation. Furthermore, the abundance and composition of the storage substances in the spore cell may also depend on dispersal strategies. In particular, a high lipid to starch ratio in mature spores has been interpreted as being a cytoplasmic adaptation to low levels of physiological activity that allow spores to tolerate longer periods after spore dispersal [13].

In this study, we assess whether ultrastructural features (plastid development, storage substances, and spore wall configuration) are associated with spore dispersal strategies. To minimize taxonomic differences, we used seven related species, all belonging to the same section of the moss genus *Orthotrichum* Hedw., in which both xerochastic and hygrochastic dispersal have been observed [18]–[20].

The genus *Orthotrichum* comprises mainly epiphytic species and is the second most speciose genus in the Iberian Peninsula (after *Bryum*) [21]. The section *Gymnoporus* Braithw. is represented by seven species in this region: two are hygrochastic and five are xerochastic. The two hygrochastic species are *O. acuminatum* H. Philib. and *O. ibericum* F. Lara & Mazimpaka; the five xerochastic species are *O. affine* Schrad. ex Brid., *O. lyellii* Hook. & Taylor, *O. speciosum* Nees, *O. striatum* Hedw., and *O. tortidontium* F. Lara, Garilleti & Mazimpaka. We have already reported the existence of two very different morphotypes in *O. affine*, which are present sometimes even within a single individual [22]; this observation suggests that external ornamentation is highly variable.

Here we present an analysis of spore ultrastructure for these seven species (including both *O. affine* morphotypes) and thus provide the first descriptions of spore internal cellular structure in this section. Using these descriptions, we assessed whether the spores of the two hygrochastic species, when compared with their xerochastic allies, show cytological characters tied to greater sensitivity and adaptation to rapid germination, namely: 1) thinner walls, especially in the sporopolenin-containing layers (exine and perine); 2) plastids with better developed thylakoids; 3) rapidly metabolized storage substances, such as starch, rather than lipids; and 4) any ultrastructural sign of precocious germination.

## Materials and Methods

For all six species (and both *O. affine* morphotypes), fresh specimens bearing mature capsules were collected at several Iberian Peninsula sites, as detailed in Table S1. All specimens were sampled in mid-mountain Mediterranean areas, with the only exception being one *O. affine* specimen (out of seven), that was collected in lower lands (Loeches, 590 m). Voucher specimens were deposited in the MAUAM herbarium at the Universidad Autonóma de Madrid, Spain. No species here analyzed are protected. In compliance with Spanish legislation, sampling did not require specific permissions as it involved sporadic collection of small quantities of specimens of non-protected plant species. All sampling locations are public lands, or public access-easements, so no permissions were required from land owners. We considered a specimen to be a single, homogeneous bryophyte patch, in accordance with the usual criteria used in bryophyte population monitoring [23].

### Light microscopy observations

The size and shape of fifty spores per capsule were examined using a Motic microscope equipped with image analysis software; spores were directly mounted in water. Bicellularity was assessed after spores were soaked in Lugol's iodine overnight to stain their cell walls. We analysed two specimens, five capsules per specimen, for each species. Sampling locations are listed in Table S1.

### Ultrastructure observations

Capsules bearing mature spores were rehydrated overnight at 4°C; fixed in 3% glutaraldehyde in Na-cacodylate buffer (0.1 M, pH 7.2); rinsed three times in the buffer; secondarily fixed in 1% osmium tetroxide; rinsed in the buffer; and dehydrated using an ethanol series. For the scanning electron microscope (SEM) observations, spores were subject to critical-point drying, sputtered with a gold coating (ca. 300 Å) and examined using a Hitachi S-3000N SEM operating at 20 kV. For the transmission electron microscope (TEM) observations, spores were placed in propylene oxide kept at 4°C and embedded in Spurr's resin [24] in seven steps. Ultrathin sections were made using a Leica Ultracut-S ultramicrotome, contrasted with uranyl acetate and lead citrate, and examined using a JEOL-JEM 1010 TEM operating at 100 kV. For the SEM observations, we analyzed one specimen per species (except in the case of *O. affine*—because of its greater variability, we studied five specimens from different locations). For the TEM observations, we analyzed at least two specimens per species. The sampling locations of the specimens used for both types of microscopy are listed in Table S1.

### Germination tests

Spore vitality was tested by culturing the spores in half-strength Murashige-Skoog basal liquid medium (pH 6.5); they were kept in a culture chamber maintained at 24°C with an 18∶6 photoperiod.

## Results

The figures showing the spore ultrastructure of the species studied are presented in alphabetical order, as follows: *O. acuminatum* (Fig. 1), *O. affine*, morphotypes I and II (Figs. 2 and 3), *O. ibericum* (Fig. 4), *O. lyellii* (Fig. 5), *O. speciosum* (Fig. 6), *O. striatum* (Fig. 7) and *O. tortidontium* (Fig. 8).

**Figure 1:**
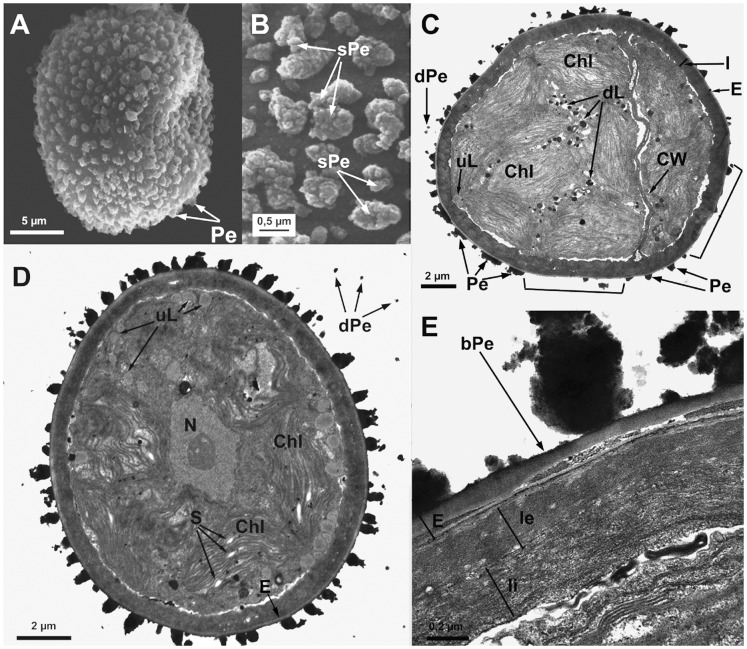
Spore ultrastructure of *Orthotrichum acuminatum*. A–B: SEM, C–E: TEM. A) Ellipsoidal spore ornamented with perine elements (verrucae and baculi). B) Detail showing primary elements (verrucae and baculi) covered with abundant papillose secondary processes dispersed across their surface. C) Bicellular spore. Note the loosely attached perine verrucae surrounding the spore. Ample areas of the spore proximal surface appear almost naked, with little sculpturing material (square brackets). Exine very thin, formed by electron-translucent, compact material. Cytoplasm dense, granular; plastids densely disposed with well developed, packed thylakoids. Note the presence of undissolved, lipidic droplets (most of them electrondense and partially dissolved) filling only a small fraction of the cytoplasm D) Unicellular spore. The inner surface of the intine appears typically undulated. Starch grains are frequent within the plastids. Note the scattered presence of undissolved, more electrontranslucent, lipidic droplets. E) Detail of the spore wall showing perine with sparse primary elements, connected by a thin basal layer and exine separating into parallel lamellae, the inner ones intermingled with intinous material (a common phenomenon in particular areas of these spores). Intine two-layered, the external layer amorphous, with granular, electrontranslucent material; the innermost more electrondense and rather fibrous. Abbreviations: bPe: basal layer of perine material; Chl: chloroplasts; CW: cell wall; dL: partially dissolved, electrondense lipidic droplets; dPe: detached perine elements; E: exine; I: intine; Ie: external layer of the intine; Ii: internal layer of the intine; N: nucleus; Pe: perine elements; sPe: perine secondary processes; S: starch grains; uL: undissolved, electron translucent lipid droplets.

**Figure 2:**
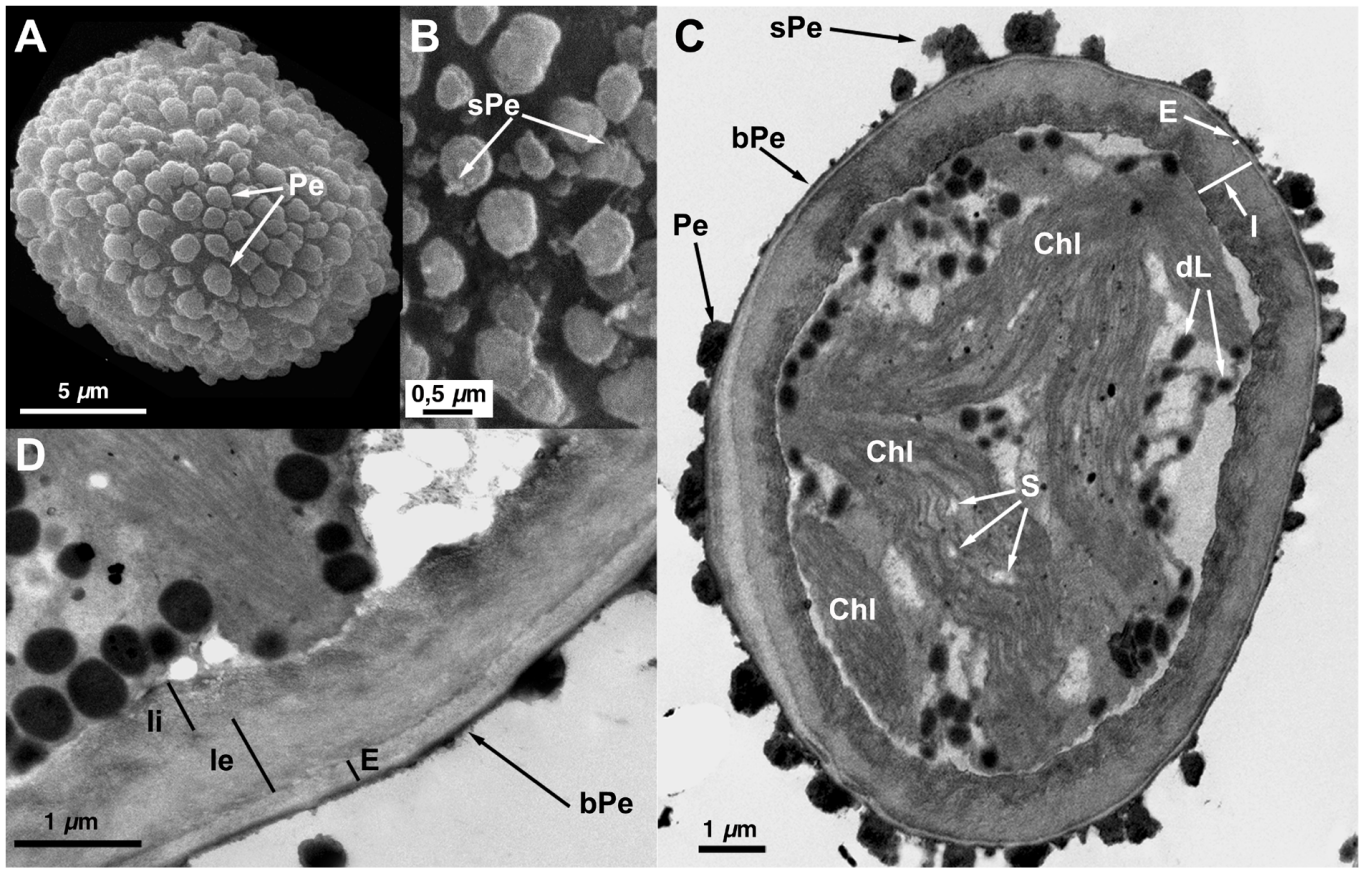
Spore ultrastructure of *Orthotrichum affine* (type I). A–B: SEM, C–D: TEM. A) Subspherical spore ornamented with irregular primary elements forming verrucae B) Detail of the spore showing primary elements covered with abundant, irregular secondary processes C) Verrucae are ornamented with abundant secondary processes. Perine elements appear connected by a relatively thick basal layer of electrondense material. Exine electrontranslucent, clearly polarized, proximally thin and compact, distally enthickened and locally separating into parallel lamellae. Plastids with well-developed thylakoids, fusiform, loosely disposed in the cytoplasm. Small starch grains present within the plastids. D) Wall structure of a spore in detail. Distal part of the spore with thick exine. Intine two-layered, outer layer granular, inner layer fibrillar, undulate, and more electrondense. Abbreviations: bPe: basal layer of perine material; Chl: chloroplasts; dL: partially dissolved, electrondense lipidic droplets; dPe: detached perine elements; E: exine; I: intine; Pe: perine elements; sPe: perine secondary processes; S: starch grains.

**Figure 3:**
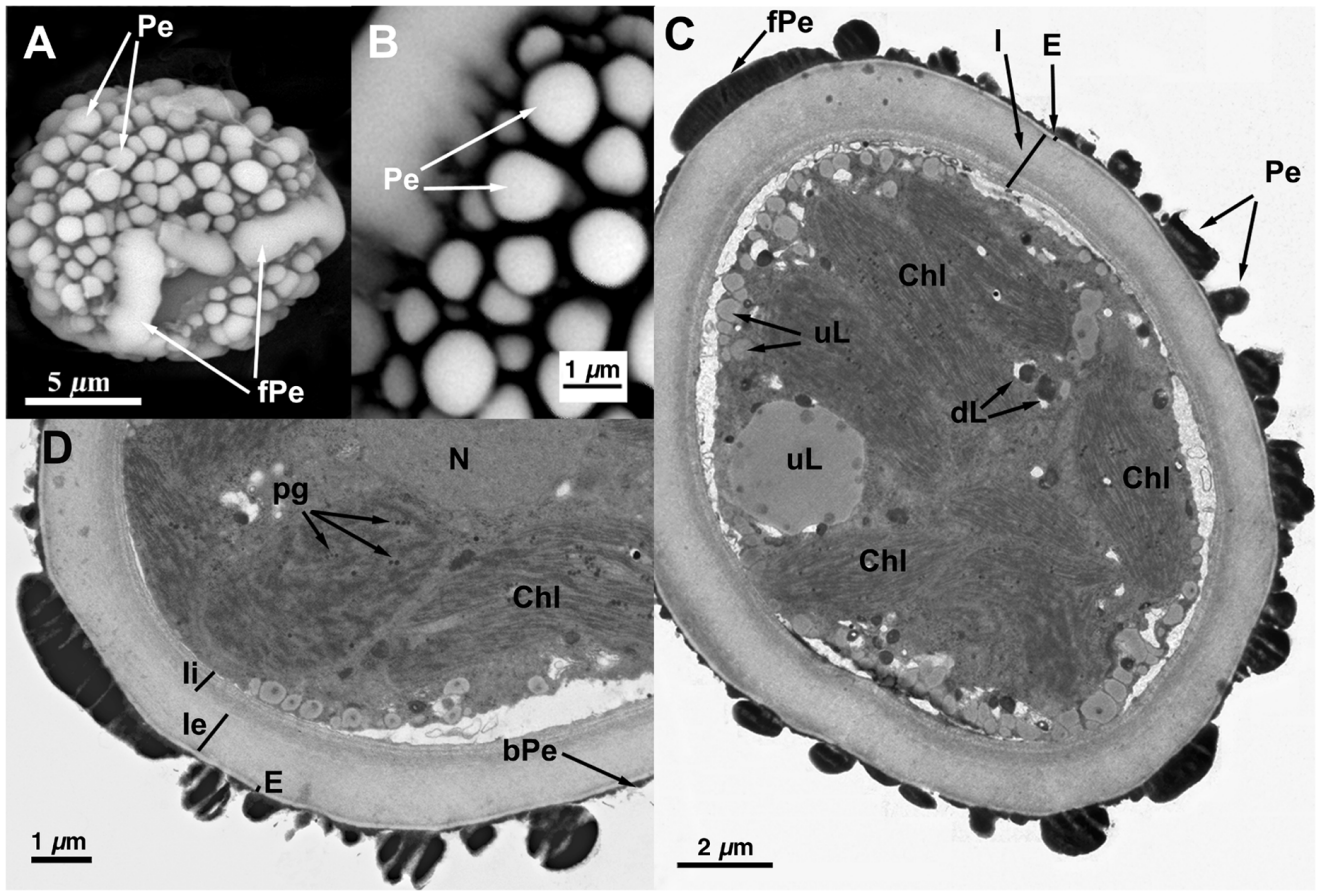
Spore ultrastructure of *Orthotrichum affine* (type II). A–B: SEM, C–D: TEM. A) Subesphericall spore. Smooth perine elements cover the spore surface; some primary elements appear fused forming extensive bands throughout the spore (a feature common in type II morphotipes of this species). B) Detail of spore ornamentation showing smooth perine elements without secondary processes. C) General view of a spore, note the smooth perine and the fused primary elements in cross section. Exine thin, of uniform thickness, without polarization. Intine electrontranslucent, bilayered. Plastids well developed. Lipidic droplets abundant, most of them medium-electrondense and undissolved. D) Detail of a spore, note the continuous basal layer of perine connecting the primary elements and the bilayered structure of the intine, the outer layer compact, the inner layer thinner, fibrillar, lamellated. Abbreviations: bPe: basal layer of perine material; Chl: chloroplasts; dL: partially dissolved, electrondense lipidic droplets; dPe: detached perine elements; E: exine; fPe: fused perine elements; I: intine; Ie: external layer of the intine; Ii: internal layer of the intine; N: nucleus; Pe: perine elements; pg: plastoglobuli; sPe: perine secondary processes; S: starch grains, uL: undissolved, electron translucent lipid droplets.

**Figure 4:**
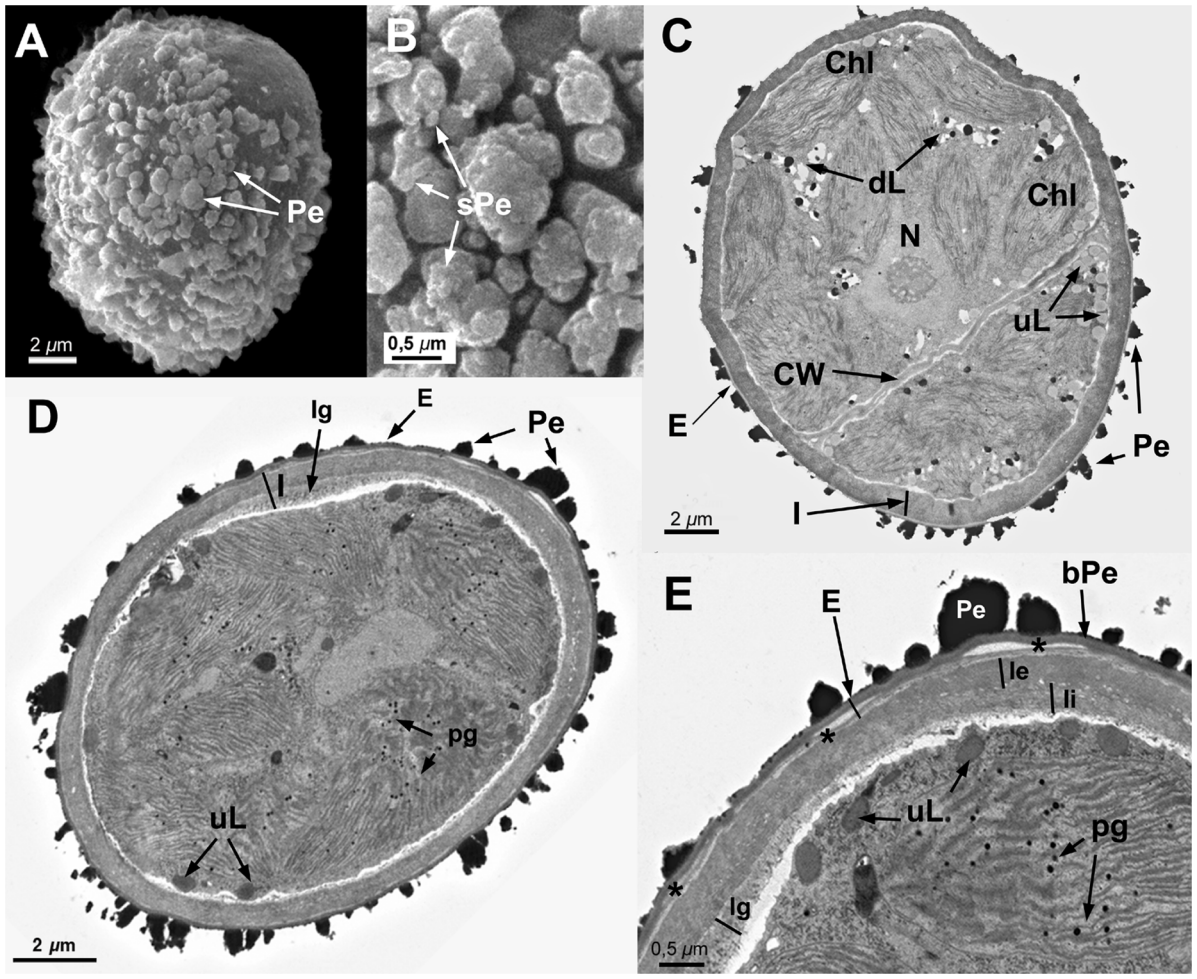
Spore ultrastructure of *Orthotrichum ibericum*. A–B: SEM, C–E: TEM. A) A subellipsoidal spore (usually slightly flattened), irregularly ornamented with perine elements, leaving large naked areas. B) Detail of the sporoderm showing perine primary elements (verrucae and baculi) covered with secondary processes (scattered papillae). C) A bicellular spore showing a sporoderm with sparse perine elements and polarized intine (proximally much thicker), and a granular cytoplasm with loosely packed chloroplasts and lipidic droplets (usually sparse in this species), some electrondense and partially dissolved, more often medium-electrondense, undissolved. D) A spore showing the sporoderm with two continuous intine layers and an occasional, internal, electrontranslucent, granular layer locally present; and exine, electrontranslucent and extensively separating into parallel lamellae. In the cytoplasm the chloroplasts appear with abundant plastoglobuli. E) Detail showing the sporoderm with a very thin basal layer connecting perine elements; the exine extensively separating into parallel lamellae (asterisks); and the intine stratification: an outermost layer amorphous, an inner granular layer, and a discontinuous granular, electrontranslucent, innermost layer. Abbreviations: bPe: basal layer of perine material; Chl: chloroplasts; CW: cell wall; dL: partially dissolved, electrondense lipidic droplets; E: exine; I: intine; Ie: external layer of the intine; Ig: innermost discontinuous intine layer; Ii: internal continuous layer of the intine; N: nucleus; Pe: perine elements; sPe: perine secondary processes; pg: plastoglobuli; S: starch grains; uL: undissolved, electron translucent lipid droplets.

**Figure 5:**
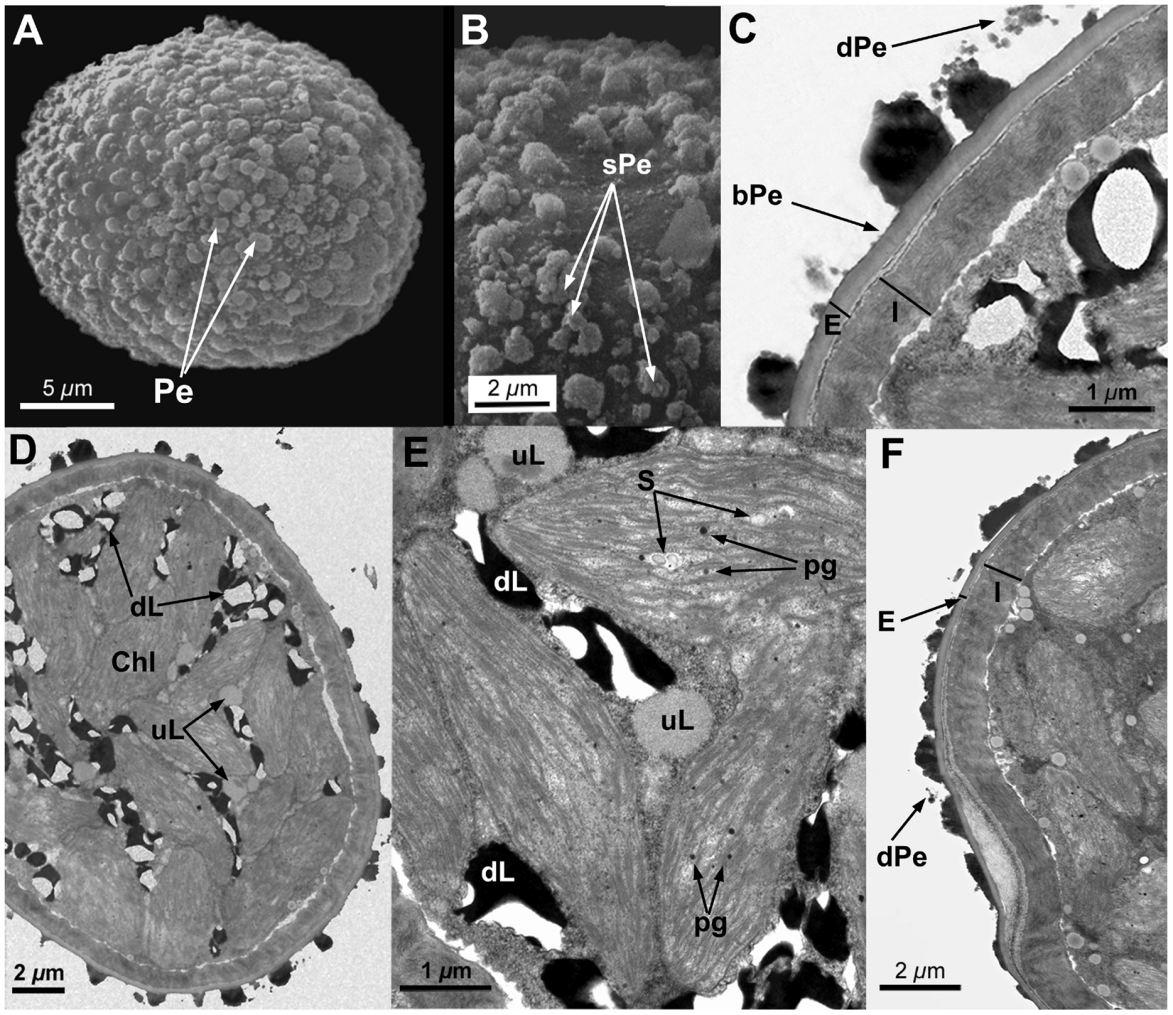
Spore ultrastructure of *Orthotrichum lyellii*. A) Subspherical spore (usually subespherical to subellipsoidal in the species) with small sized verrucae scattered throughout the spore surface. B) Detail of the perine elements with abundant granular secondary processes over the surface of verrucae. C) Detail of the sporoderm structure showing electrondense perine and a very thin basal layer of electrondense material joining verrucae (frequent in the species). Note also the aggregations of detached secondary elements. The spore shows a thick electrontranslucent exine locally separated into parallel lamellae, layers of exine lamellae alternating electron translucent and opaque material. Intine electrontranslucent, one layered, fibrillar, variable in thickness, inner surface of the intine undulate. D) Granular cytoplasm bearing chloroplasts with well developed thylakoids. Lipid droplets abundant, of two different types, some medium-electrondense undissolved, most electrondense and partially or almost totally dissolved. E) Detail of the cytoplasm, note the starch granules within the chloroplast (a sporadic feature in the spores of this species. F) Detail of sporoderm that shows a large gap filled with electrontranslucent material between intine and exine that ocasionally appears in some of the spores. Abbreviations: bPe: basal layer of perine material; dPe: Chl: chloroplasts; CW: cell wall; dL: partially dissolved, electrondense lipidic droplets; dPe: detached perine elements; E: exine; I: intine; Ie: external layer of the intine; Ii: internal layer of the intine; N: nucleus; Pe: perine elements; sPe: perine secondary processes; S: starch grains; uL: undissolved, electron translucent lipid droplets.

**Figure 6:**
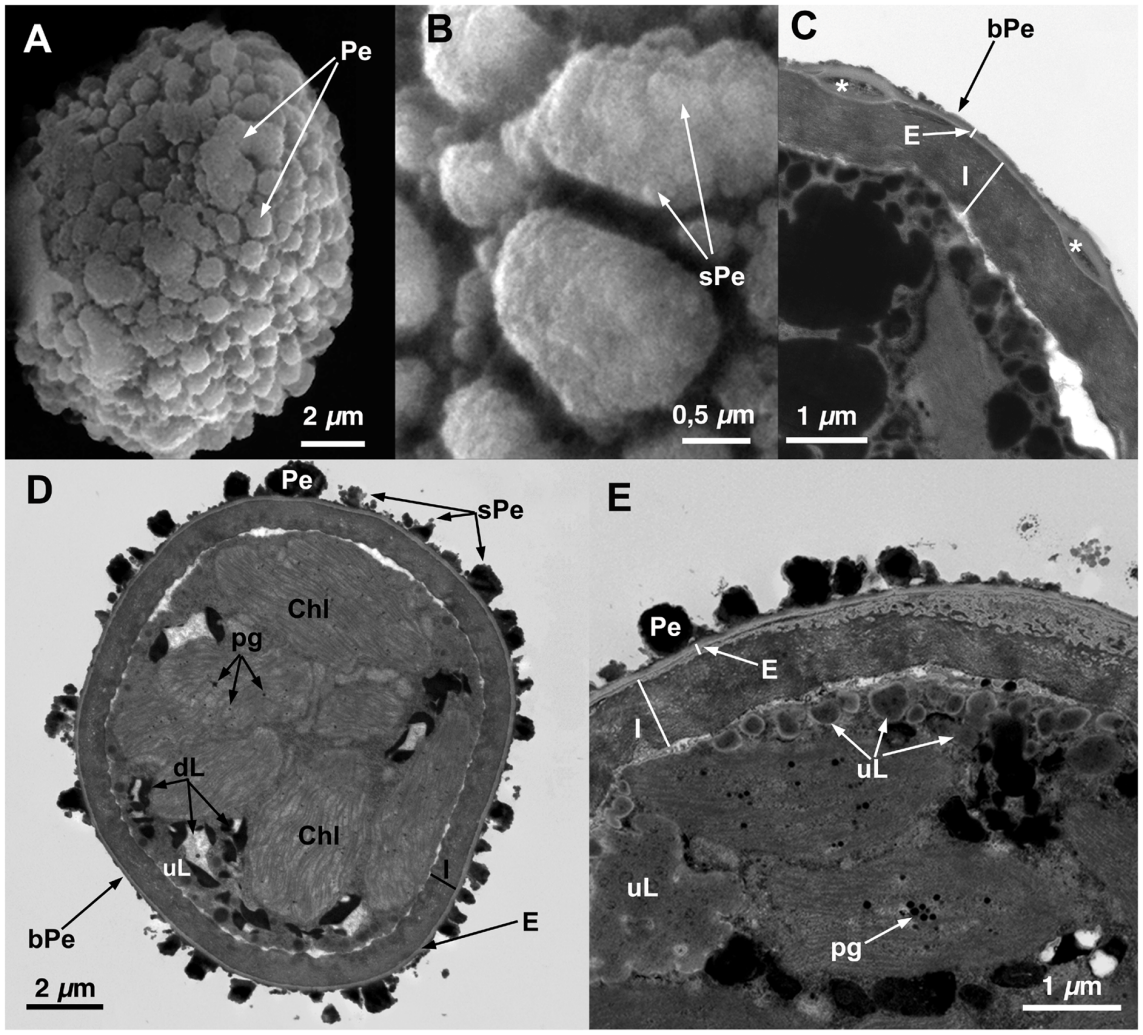
Spore ultrastructure of *Orthotrichum speciosum*. A) Subspherical spore. Verrucae irregularly scattered, sometimes fused into heterogeneous units, variable in shape (spike-like to rounded) and size. B) Detail of the perine, granular secondary processes abundant throughout the basal layer and the verrucae. C) Detail of the sporoderm. Perine electrondense, elements connected by a electrondense basal layer. Granular secondary processes abundant throughout the basal layer and the verrucae. Exine thin, electrontranslucent, locally separating into parallel lamellae, with occasional conspicuous gaps filled with electrondense material (asterisks). Intine bilayered, outer layer fibrillar-granular, inner layer fibrillar, undulate. D) General view of a spore. Cytoplasm granular. Chloroplasts with well developed thylakoids. Lipid droplets abundant, of two different types. Some medium-electrondense, electrondense droplets more abundant. E) Chloroplasts with numerous plastoglobuli. Contact between exine and intine with expanded and labyrinth-like intrusions. Abbreviations: bPe: basal layer of perine material; Chl: chloroplasts; dL: partially dissolved, electrondense lipidic droplets; dPe: detached perine elements; E: exine; I: intine; Pe: perine elements; sPe: perine secondary processes; S: starch grains; uL: undissolved, electron translucent lipid droplets.

**Figure 7:**
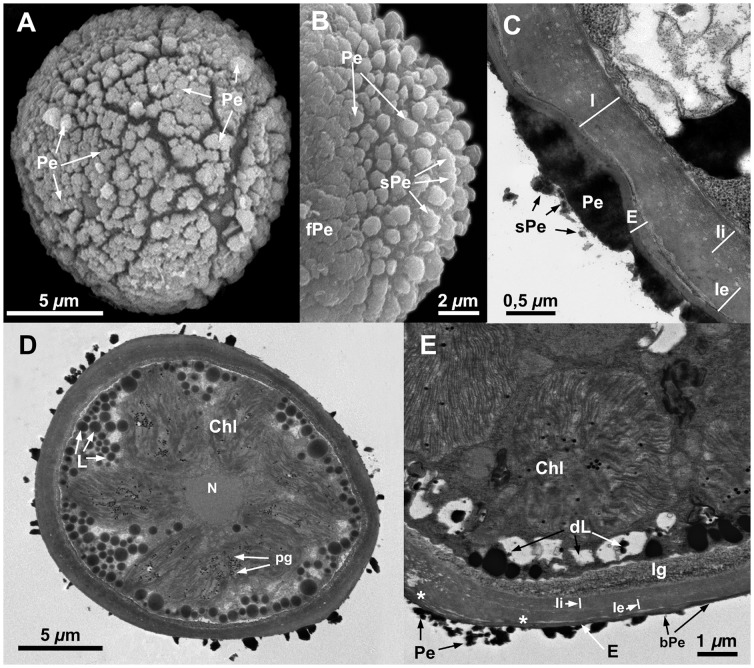
Spore ultrastructure of *Orthotrichum striatum*. A–B: SEM, C–E: TEM. A) A subellipsoidal spore, ornamented with dense, irreglarly fused, perine elements. B) Perine primary elements (verrucae), sometimes fusing into a plate, and covered with abundant secondary processes. C) Detail showing the sporoderm stratification: fused perine verrucae; a thick exine separating into parallel lamellae (in this species this happens in extense areas of the sporoderm); and an intine with two continuous layers, the outermost granular and the innermost fibrillar. D) Spore showing a sporoderm irregular in thickness; and a cytoplasm filled with copious lipidic droplets (some partially dissolved, most undissolved) and chloroplasts with abundant plastoglobuli. E) Detail showing sporoderm with perine verrucae connected by an electrondense basal layer, exine separating into parallel lamellae (asterisks) and intine with an inner thickening of granular material, additional to its two continuous layers. In the cytoplasm, electrondense, partially dissolved lipidic droplets, and chloroplasts with well developed thylakoids and plastoglobuli, are observed.

**Figure 8:**
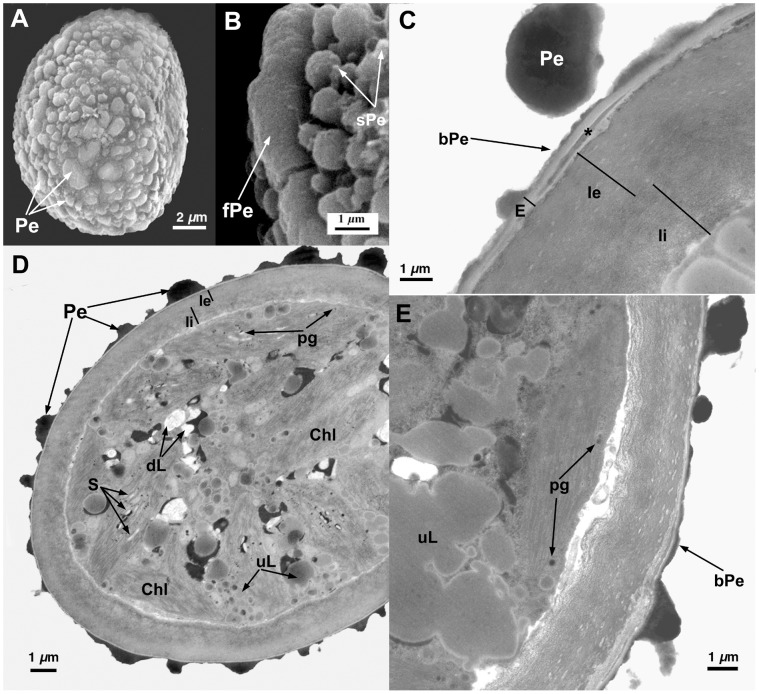
Spore ultrastructure of *Orthotrichum tortidontium*. A–B: SEM, C–E: TEM. A) Spore subellipsoidal. Verrucae large, irregular in shape and size. B) Primary elements irregularly fused, occasionally forming large bands. Secondary processes scarce, irregularly present on the verrucae. C) Perine electrondense, elements connected by an electrondense basal layer. Exine thick, electrontranslucent, locally separating into parallel lamellae (asterisk). Intine electrontranslucent, thick, two layered. D) Cytoplasm granular. Chloroplasts with well developed thylakoids, often with starch. Abundant lipid droplets of two different types, some electrontranslucent, undisolved, the others electrondense and partially or almost totally dissolved. E) Detail of sporoderm structure showing two layered intine, outermost layer granular, innermost layer granular-fibrillar. Abbreviations: bPe: basal layer of perine material; Chl: chloroplasts; CW: cell wall; dL: partially dissolved, electrondense lipidic droplets; dPe: detached perine elements; E: exine; fPe: fused perine elements; I: intine; Ie: external layer of the intine; Ii: internal layer of the intine; N: nucleus; Pe: perine elements; sPe: perine secondary processes; S: starch grains; uL: undissolved, electron translucent lipid.

All seven species have subspherical to ellipsoidal spores, often flattened at one or both poles (e.g., Figs. 1A and 6A), which ranged in size from 12 to 38 µm. No external apertures were observed.

Under the TEM, the sporoderm was found to have the three distinct layers that are characteristic of mosses, as defined by McClymont & Larson [25]: the perine, the exine, and the intine. Spore external ornamentation consists only of perine. This layer is composed of electron-opaque materials, discontinuous, characterized by irregular protuberances and verrucae, and often ornamented by secondary processes (sometimes absent in some *O. affine* spore morphotypes [22], see also Figs. 3A, 3B). A very thin continuous basal layer (discontinuous only in *O. ibericum*) is present (e.g. Figs. 8C, 8E). The external elements of the perine came off easily, and therefore the spores were often partially or even completely free of verrucae (Figs. 1C and 4A); others, in contrast, had surfaces almost entirely covered in dense ornamentation.

The exine is electron-translucent and homogeneous. It is usually thin and unbroken (e.g., Figs. 1D, 2C, and 6D) and is sometimes locally divided up into parallel lamellae, which are interspersed with intine-like material (Figs. 1E, 4E, 5F, 6C, 7C, and 8C). *O. striatum* (Fig. 7C) and *O. ibericum* (Fig. 4E) has the most extensively developed lamellation, which left gaps that were sometimes but not always filled with granular or amorphous material. The remaining species demonstrate a rather localized lamellation. The intine typically consists of two continuous layers, usually fibrillar (e.g., Figs. 1E, 2D, 7C, and 8C). *O. lyelli*, however, presents a unilayered intine (Fig. 5C). Occasionally, *O. ibericum* and *O. striatum* spores show some inner thickenings that form a discontinuous third layer (Figs. 4E and 7E).

The thickness of the sporoderm is highly variable, and thickness values overlap a great deal among species (Table 1). Spore shape and sporoderm structure are both rather irregular. The spore demonstrates polar organization: the distal and proximal poles were always distinguishable in either the sporoderm or the cytoplasm (and sometimes in both). Spores are usually unicellular and only rarely bicellular (1–3% of the spores), except in *O. ibericum* (Fig. 4C) and *O. acuminatum* (Fig. 1C), where 10–20% of spores are bicellular.

**Table 1:** Main spore features of each species.

	Size	Sporoderm			Cytoplasm	Notable features
		Perine: thickness, element density, and occurrence of a basal layer	Exine: thickness and lamellation	Intine: thickness and layering	Lipidic droplets	
***O. acuminatum***	22–25 (15–30) µm	0.59 (0.3–0.8) µm, sparse, basal layer present	0.07–0.14 µm, locally lamellated	0.94 (0.6–1.4) µm, bilayered	Scarce	Spores often bicellular (ca. 20%)
***O. affine: Type I***	18–20 (14–22) µm	0.61 (0.4–0.8) µm, sparse, basal layer present	0.09 (0.04–0.16) µm, polarized, locally lamellated	0.99 (0.7–1.45) µm, bilayered	Abundant	Spores rarely bicellular (<1%)
***O. affine: Type II***	18–20 (14–22) µm	0.73 (0.6–1.0) µm, sparse, basal layer present	0. 07 (0.06–0.1) µm, locally lamellated	1.38 (1.10–1.55) µm, bilayered	Very abundant	Spores rarely bicellular (<1%); perine elements smooth, irregularly fused into ridges
***O. ibericum***	16–18 (12–25) µm	0.57 (0.2–1.2) µm, very sparse, very thin discontinuous basal layer	0.1 (0.03–0.18), extensively lamellated	0.71 (0.36–1.32) µm, bilayered	Scarce	Spores often bicellular (ca. 10%); intine occasionally 3-layered
***O. lyellii***	18–21 (12–30) µm	0.38 (0.15–0.6) µm, sparse, basal layer present	0.20 (0.08–0.30) µm, locally lamellated	0.71 (0.21–1.48) µm, unilayered	Abundant	Spores occasionally bicellular (2–3%)
***O. speciosum***	18–20 (12–25) µm	0.72 (0.4–1.2) µm, medium dense, basal layer present	0.08 (0.06–0.11) µm, locally lamellated	0.86 (0.56–1.05) µm, bilayered	Abundant	Exine with occasional labyrinth-like intrusions
***O. striatum***	18–21 (13–38) µm	0.37 (0.08–1.05) µm, dense, basal layer present	0.14 (0.06–0.2) µm, extensively lamellated	1.08 (0.62–1.57) µm, bilayered	Abundant	Intine occasionally 3-layered
***O. tortidontium***	16–18 (15–23) µm	0.66 (0.43–1.02) µm, medium dense, basal layer present	0.06 (0.03–0.11) µm, locally lamellated	0.94 (0.74–1.43) µm, bilayered	Abundant	

The most common range (for spore sizes) or mean value (for layer thickness) is indicated first; the maximum range observed is then indicated in parentheses.

The cytoplasm contains a large number of more or less fusiform plastids (e.g., Figs. 2C, 3C, and 8D). Thylakoids are well developed within the plastids, and they frequently contain small grains of starch (e.g., Fig. 1D). Droplets of plastoglobuli formed by electron-dense material are also common within plastids and were observed in all species (e.g., Figs. 4E and 7D).

Globular lipid droplets varying in size are also abundant in the cytoplasm; some of them are filled with partially or almost entirely solubilized electron-dense material (e.g., Figs. 1C, 5D, and 5E), while others are more or less translucent (e.g., Figs. 6C and 8E). According to Olesen & Mogensen [26], these differences mean that the lipids differ in their solubility. The lipid droplets are abundant in most of the species (e.g., Figs. 2C, 5D, and 7D), with the exception of *O. acuminatum* (Fig. 1D) and *O. ibericum* (Fig. 4D), in which they are relatively scarce.

In all seven species, most spores were able to germinate within a short time period, so we assume the healthy status of the populations examined. The figure legends and Table 1 provide more detailed observations on each species.

## Discussion

### Taxonomic significance

The particularities of the exine and the differences in intine stratification that we observed are important bryophyte features that may be of systematic significance. All seven species studied here showed lamellation in at least some areas of the exine. There was some interspecific variation: lamellation is more extensive in *O. ibericum* and *O. striatum* but occurs only occasionally in the other species.

This result is significant because the presence of a lamellated exine in mature spores is thought only to occur in Div. Marchantiophyta (liverworts) among all land plants and, on this basis alone, some Ordovician fossil spores have been tentatively ascribed to this bryophyte group [4]. Other studies in addition to ours have noted that locally lamellated exine occurs in the mature spores of several moss genera [11], [14], [15], which shows that this phenomenon might not be so exceptional in mosses after all. As far as we know, the extent of lamellation is different in the two groups of bryophytes; it seems to occur in restricted areas of the exine in mosses, whereas in liverworts the entire exine may be lamellated. In addition, the multilamellated exine of liverworts seems to be structurally different to that of mosses. Clearly, a detailed study of the lamellation patterns in bryophytes is needed to determine if this feature is indeed unique to the liverworts.

The presence of irregular, labyrinth-like intrusions into the exine (Fig. 6E), a feature never previously described, occurred only in *O. speciosum*. Both the significance and the frequency of these intrusions in mosses are unknown.

Similarly, intine stratification is relatively unexplored in mosses. In our study species, the intine was usually found to be bilayered, although *O. lyellii* intine has only one layer and *O. ibericum* and *O. striatum* spore walls occasionally have a third intine layer. Within the species of this section, intine structure is a fairly consistent character. It has also been pointed out that this character may possibly have taxonomic value in other moss genera [11], [15], although further studies are needed to exclude the possibility that intine stratification is dependent on environmental or developmental conditions. Recently, other previously unknown sporoderm features, such as intine protrusions, have been found in the moss genus *Ptychomitrium* [15]. These findings suggest that, in order to assess the possible systematic or developmental significance of the features mentioned above, TEM studies need to be extended to include a wider taxonomic spectrum of bryophytes.

In contrast to internal characters, size and external sculpturing, the two most studied spore characters in mosses, were not significantly different among our study species. The size range we observed matches those given in the most commonly used descriptions of the genus *Orthotrichum* (e.g. [18]–[20]), and although *O. acuminatum* tends to have larger spores, there was nonetheless a high degree of overlap with other species.

Spore ornamentation depends only on perine elements (primary and secondary). There are some interspecific differences in their density, but intraspecific variation also occurs within all species. It is notable that, in *O. affine*, reported intraspecific heterogeneity in the external morphology of the two morphotypes [22] is greater than the interspecific diversity observed among other species of the subgenus. Besides, no TEM-observed characters provide independent support for these external differences, which is not surprising as both morphotypes may even coexist within the same plant [22].

Our results corroborate the common occurrence of locally lamellated exine in moss spores and underscore the existence of infrageneric variability in intine structure. We also highlight that, in order to confirm character findings obtained with light microscopy and SEM, it is important to complement external observations with the study of internal ultrastructure using TEM.

### Functional significance

Spores in the two hygrochastic species, *O. acuminatum* and *O. ibericum*, show a tendency to accumulate smaller quantities of lipids and to have higher proportions of bicellular spores. Multicellular spores occur constantly in some species of epiphytic mosses [27], [28], but the species analyzed here are typically unicellular. As a result, we interpret internal tabications as being a sign of precocious germination. However, contrary to our expectations, we did not find any clear-cut differences between the spores of xerochastic and hygrochastic species in Iberian representatives of *Orthotrichum* in the sect. *Gymnoporus*. Overall, most spore cytological characters were similar: sporoderm structure and thickness, degree of plastid development, and the main storage substances used.

In all six species, the storage substances in spores are mainly lipids, which were usually present in large quantities. They are considered to be an adaptation that helps spores withstand environmental stress [13]. On the other hand, spores have thin sporoderms (especially the exine and perine layers) and well-developed plastids, two traits that are regarded as typical of sensitive or “green spores” and that are is usually linked to a rapid germination strategy [10], [13]. Indeed, the most distinctive spore features of both hygrochastic species (a higher frequency of precocious germination signs and lesser quantities of lipids) could also be interpreted as adaptations to immediate post-release germination, a strategy where a high resistance over prolonged periods would not be necessary.

In short, the idea that differences in spore sensitivity underlie hygrochastic strategies in the *Orthotrichum* sect. *Gymnoporus* is only partially supported by our ultrastructural observations: in a context of general cytological similarity, hygrochastic species show some differences in the amount of lipids stored and the proportion of bicellular spores. Further quantitative studies would be needed for a statistical verification of these differences. Apart from spore sensitivity, other hypotheses have been invoked to explain the hygrochastic strategy. For instance, the advantage of this strategy is clear when water is a significant dispersal vector [29], [30] or when persistently wet conditions seriously limit xerochastic dispersal, as is the case for bryophytes growing in tropical rainforests [16]. In other cases, hygrochasy is thought to engender temporal constraints, only resulting in diaspore release when conditions for germination are most favorable. Thus, in vascular plants, it has been described in many species found in arid environments (e.g. [31]–[34]). In this cases hygrochasy would also constrain dispersal distance [35], [36]. Such constraints are often considered to have negative effects because they limit a plant's ability to colonize new areas. Nevertheless, some authors suggest that dispersal constraints can positively affect species survival in patchy environments; this may occur in desert plants (e.g. [33], [37]), and, interestingly, in alpine environments. Pufal & Garnock-Jones [38], who have studied hygrochastic *Veronica* species in the mountains of New Zealand, suggest that these plants utilize a safe-site strategy: in patchy environments, a space-based dispersal constraint may be advantageous because seeds are forced to land in appropriate patches where their survival is favored. Epiphytic bryophyte communities also grow in patchy and dynamic habitats [39], [40]. Therefore, a safe-site strategy may affect positively the establishment success of some of these species, thus favoring the development of hygrochastic syndromes also in dry, Mediterranean environments. This hypothesis is supported by the fact that peristomial reduction (one of the morphologic modifications related to hygrochasy in mosses) is more common in epiphytic species [41]–[43]. Further research is needed to assess the relationship between hygrochasy and a safe-site strategy. Here, in mosses, and particularly in the genus *Orthotrichum*, it would be worthwhile to investigate possible differences in the spatial aggregation patterns and ecological and physiological optima of xerochastic *versus* hygrochastic species.

## Concluding Remarks

The spores of xerochastic and hygrochastic species show only subtle ultrastructural differences. A lower abundance of lipids and a higher proportion of bicellular spores seem to be associated with hygrochasy. We have not observed any clear-cut ultrastructural character consistent with a greater sensitivity in spores with this dispersal strategy. We discuss the relevance of these findings, and hypothesize that hygrochasy in Mediterranean environments might respond to a spatial, safe-site strategy rather than to an avoidance of dry, unfavorable periods. However, the real effects of the differences observed need to be assessed under natural conditions.

We observed some previously undescribed spore features, namely related to the wall layers. The taxonomic or developmental significance of the intine layers as well as protrusions and irregularities present in some of these species needs to be assessed in a wider context by sampling moss taxa with contrasted life-history traits along environmental gradients. We underscore the effectiveness of transmission electron microscopy as tool for identifying new characters and emphasize that our understanding of moss spore ultrastructure remains incomplete.

## Supporting Information

Table S1 Specimens examined.
(DOC)
