# Supplementary material for: Does Spore Ultrastructure Mirror Different Dispersal Strategies in Mosses? A Study of Seven Iberian Orthotrichum Species
Source: PLoS One. 2014 Nov 20;9(11):e112867. doi: 10.1371/journal.pone.0112867 (PMC4239030; doi:10.1371/journal.pone.0112867)
Supplement: Table S1 — Specimens examined. (DOC) [file pone.0112867.s001.doc]

Table S1: Localities of the specimens examined*

| ***Orthotrichum acuminatum* H.Philib.** | | |
| --- | --- | --- |
| Ávila, Poyales del Hoyo. 40º 11’N, 5º 11’W, 600-700 m | *B. Estébanez & N.G. Medina*. 7 Jun. 2007 | SEM, TEM |
| Ávila, Serranillos. 40º 21’N, 4º 54’W, 1198 m | *B. Estébanez & N.G. Medina*. 7 Jun. 2007 | TEM |
| Madrid, Manzanares El Real. 40º 43’N, 3º 53’W, 900 m | *N.G. Medina*. 30 Jul. 2011 | SS, Bi, G |
| ***Orthotrichum affine* Schrad. ex Brid.** | | |
| Soria, Cañón del Río Lobos. 40º 43’N, 3º 4’W, *ca* 1000 m | *B. Estébanez & F. Lara*. 9 Oct. 2007 | SEM, TEM |
| Guadalajara, El Cardoso. 41º 5’N, 4º 40’W  1300-1450 m | *B. Estébanez & N.G. Medina*. 8 Jun. 2007 | SEM, TEM |
| Madrid, Loeches. 40º 23’N, 3º 28’W, 590 m | *D. Cortés*. 24 Oct. 2003 | SEM |
| Almería, Sierra Alhamilla. 37º 0’N, 2º 22’W, 800-1000 m | *N.G. Medina R. Medina & V. Mazimpaka.* 22 Jul. 2006 | SEM |
| Ciudad Real, El Viso del Marqués. 38º 26’N, 3º 43’W,800 m | *B. Estébanez, N.G. Medina & R. Medina.* 15 Jun. 2006 | SEM |
| Ávila, Serranillos. 40º 21’N, 4º 54’W, 1198 m | *B. Estébanez & N.G. Medina*. 7 Jun. 2007 | SEM, TEM, G |
| Ávila, Serranillos. 40º 21’N, 4º 54’W, 1198 m | *B. Estébanez.* 10 Jun. 2012 | SS, Bi |
| ***Orthotrichum ibericum* F.Lara & Mazimpaka** | | |
| Ávila, Serranillos. 40º 21’N, 4º 54’W, 1198 m | *B. Estébanez & N.G. Medina*. 7 Jun. 2007 | SEM, TEM |
| Ávila, Serranillos. 40º 21’N, 4º 54’W, 1198 m | *B. Estébanez & N.G. Medina*. 3 Jul. 2011 | SS, Bi, G |
| Ávila, Poyales del Hoyo. 40º 11’N, 5º 11’W, 600-700 m | *B. Estébanez & N.G. Medina*. 7 Jun. 2007 | TEM |
| ***Orthotrichum striatum* Hedw.** | | |
| Guadalajara, El Cardoso. 41º 5’N, 4º 40’W, 1300-1450 m | *B. Estébanez & N.G. Medina*. 8 Jun. 2007 | SEM, TEM |
| Ávila, Serranillos. 40º 21’N, 4º 54’W, 1198 m | *B. Estébanez & N.G. Medina*. 3 Jul. 2011 | G |
| Ávila, Serranillos. 40º 21’N, 4º 54’W, 1198 m | *B. Estébanez.* 10 Jun. 2012 | SS, Bi, TEM |
| ***Orthotrichum speciosum* Nees** | | |
| Guadalajara, El Cardoso. 41º 5’N, 4º 40’W, 1300-1450 m | *B. Estébanez & N.G. Medina*. 08 Jun. 2007 | TEM |
| Soria, Cañón del Río Lobos. 41º 44’N, 3º 4’W, *ca*1000 m | *B.Estébanez & F. Lara*. 9 Oct. 2007 | SS, Bi, SEM, TEM |
| Soria, Montejo de Tiermes, 41º24’14”N, 3º12’34”W, 1176 m | N.G. Medina. 25 May 2010 | G |
| ***Orthotrichum lyellii* Hook. & Taylor** | | |
| Madrid, Hoyo de Manzanares. 40º 37’N, 3º 55’W, *ca* 1130 m | *F. Lara*. 12 Oct. 2007 | SEM, TEM |
| Cáceres, Garganta la Olla. 40º7'N, 5º47'W, 715 m | *A. Ruiz Herranz*, 1 May 2012 | SS, Bi, G |
| ***Orthotrichum tortidontium* F.Lara, Garilleti & Mazimpaka** | | |
| Soria, Cañón del Río Lobos. 41º 44’N, 3º 4’W, *ca*1000 m | *B. Estébanez & F. Lara*. 9 Oct. 2007 | SS, Bi, SEM, TEM |
| Guadalajara: Valtablado del Río, 40º42’16”N, 2º24’38”W, 1008 m | *N.G. Medina*. 18 Jul. 2010 | G |

*All localities in Spain
